# Alterations in deglutition in children with congenital Zika virus syndrome

**DOI:** 10.1590/2317-1782/20212021270

**Published:** 2023-01-06

**Authors:** Débora Rios, Mino Rios, Ana Caline Nóbrega, Lia Bernadeth de Oliveira, Daniel Vaz, Henrique Sales, Breno Lima de Almeida, Leticia Serra Lopes, Isadora Cristina de Siqueira, Rita Lucena

**Affiliations:** 1 Programa de Pós-graduação em Medicina e Saúde, Faculdade de Medicina da Bahia, Universidade Federal da Bahia - UFBA - Salvador (BA), Brasil.; 2 Departamento de Psicologia, Universidade do Estado da Bahia - UNEB - Salvador (BA), Brasil.; 3 Departamento de Fonoaudiologia, Instituto de Ciências da Saúde, Universidade Federal da Bahia - UFBA - Salvador (BA), Brasil.; 4 Centro de Prevenção e Reabilitação do Estado da Bahia - Salvador (BA), Brasil.; 5 Instituto Gonçalo Moniz, Fundação Oswaldo Cruz - Fiocruz Salvador, (BA), Brasil.

**Keywords:** Zika Virus, Microcephaly, Swallowing, Swallowing Disorder, Swallowing Sounds, Doppler Effect, Vírus Zika, Microcefalia, Deglutição, Desordem da Deglutição, Sons da Deglutição, Efeito Doppler

## Abstract

**Purpose:**

To characterize swallowing in children with congenital Zika virus syndrome in comparison to typical children.

**Methods:**

This cross-sectional study enrolled 45 children diagnosed with congenital Zika virus syndrome and 45 others with typical development. Swallowing was evaluated through clinical feeding evaluations Protocolo de Avaliação Clínica da Disfagia Pediátrica and using acoustic swallowing parameters (Doppler sonar).

**Results:**

The mean age of children with congenital Zika virus syndrome was 26.69 ± 4.46 months and the mean head circumference was 29.20 ± 1.98 cm. Moderate/severe oropharyngeal dysphagia was found in 32(71.1%) of the children with congenital Zika virus syndrome. Significant differences were found between the groups on clinical evaluation: Children with congenital Zika virus syndrome presented insufficient lip closure 42(93.3%) and altered tonus of the tongue 35(77.8%) and cheeks 34(75.6%). In the children in the comparison group, only 6(13.3%) presented insufficient lip closure and 1(2.2%) had inadequate tongue posture. Changes during swallowing with liquid and spoonable food were not observed in the comparison group. When liquid/food was offered, affected children presented difficulties in sipping movements 14(77.8%) and lip/spoon contact 35(75%). The presence of residual food in the oral cavity after swallowing 38(86.4%) and clinical signs indicative of laryngotracheal penetration/aspiration, such as coughing, gagging and/or labored breathing, were also notable. No differences were found between the groups with regard to the acoustic parameters evaluated instrumentally.

**Conclusion:**

Children with congenital Zika virus syndrome present alterations in the oral phase of swallowing, as well as clinical signs indicative of pharyngeal phase impairment.

## RESUMO

**Objetivo:**

Caracterizar a deglutição das crianças com síndrome congênita do Zika vírus e comparar com crianças típicas.

**Método:**

Estudo de delineamento transversal, com 45 crianças diagnosticadas com síndrome congênita do Zika vírus e 45 típicas. A deglutição foi avaliada por meio de avaliações clínicas da alimentação através do Protocolo de Avaliação Clínica da Disfagia Pediátrica e dos parâmetros acústicos da deglutição (sonar Doppler).

**Resultados:**

A idade média das crianças com síndrome congênita do vírus Zika foi de 26,69 ± 4,46 meses e o perímetro cefálico médio foi de 29,20 ± 1,98 cm. Disfagia orofaríngea de moderada a grave foi observada em 32(71,1%) das crianças com síndrome congênita do Zika vírus, ao contrário do grupo comparação que não revelou alterações na deglutição. Nas crianças com síndrome congênita do Zika vírus foram verificados lábios entreabertos 42(93,3%) e tônus alterado em língua 35(77,8%) e bochechas 34(75,6%). Nas crianças do grupo comparação apenas 6(13,3%) apresentaram fechamento labial insuficiente e 1(2,2%) postura de língua inadequada. Alterações durante a deglutição com líquido e alimento pastoso não foram observadas no grupo comparação. Durante a oferta do alimento ocorreram dificuldades no movimento de sorver 14(77,8%), na captação da colher 35(75%), resíduo em cavidade oral 38(86,4%) e sinais clínicos indicativos de penetração/aspiração laringotraqueal como tosse, engasgo e respiração com esforço. Não foram encontradas diferenças nos parâmetros acústicos da avaliação instrumental.

**Conclusão:**

as crianças com síndrome congênita do Zika vírus têm alterações nas fases oral e faríngea da deglutição, com sinais clínicos indicativos de penetração/aspiração laringotraqueal quando comparadas a crianças típicas.

## INTRODUCTION

Eating and swallowing are developmental phenomena that involve highly complex interactions, beginning in embryological and fetal periods and continuing throughout early childhood^(1)^. Swallowing, a function that requires the involvement of neuronal networks coordinated by the cortical regions and the brainstem, when compromised, can place the airways at risk of obstruction, and may lead to inadequate nutritional intake^(2,3)^.

Observational studies in children with Congenital Zika virus syndrome (CZS) have shown high frequencies of motor, cognitive, language and dietary impairment^(4,5)^. The observed difficulties in eating appear to be linked to severely impaired sensorimotor development^(6)^. In affected children, eating problems have mainly been identified in the oral and pharyngeal phases of swallowing, related to dystonic tongue movements, inadequate lingual posture and shortened labial and lingual frenulum, in addition to reduced pharyngeal sensitivity^(7-9)^. These alterations can lead to an increased risk of aspiration, malnutrition and dehydration^(10)^.

Dysphagia is investigated in pediatric populations through the clinical assessment of food consumption and instrumental examinations that assist in defining the physiological status of swallowing^(11)^. As an affordable alternative to instrumental assessments, sounds and vibrations during swallowing have been investigated using a stethoscope or acoustic detectors (microphones/accelerometers)^(12)^. In light of this consideration, the present study employed Sonar Doppler, an easy and inexpensive non-invasive procedure^(13)^ that has been recently adopted to assess swallowing through the evaluation of acoustic parameters^(14)^.

Considering that CZS presents as a wide spectrum of manifestations and that oropharyngeal dysphagia can increase the risk of respiratory infection, inhibit weight gain, and stunt growth, the findings of this study may help guide intervention strategies to improve the quality of life of in children with CZS. Therefore, the present study aimed to characterize swallowing function in children with CZS through clinical evaluations of feeding and acoustic swallowing parameters (duration, frequency and amplitude) in comparison to typical children.

## METHODS

### Participants

This cross-sectional study assessed swallowing in children with CZS and controls through age- and sex-matched comparisons. Children with CZS included in this study were born between November 2015 and February 2016 and were recruited between January 2017 and August 2018. The sample consisted of children of both sexes aged between 12 and 32 months who were born during the first Zika virus outbreak in Brazil. Children with confirmed or highly probable CZS were included, i.e., those presenting clinical features such as microcephaly, craniofacial disproportion, hearing and eye abnormalities associated with neuroimaging characteristic of CZS according to the criteria established by França et al.^(15)^. All parents voluntarily signed a term of informed consent. This study was approved by the Institutional Review Board. For comparison purposes, children with typical development (controls) were recruited from two daycare centers located in the same city. All control individuals were born to term (>37 weeks) and had no history of eating difficulties or abnormal neurological conditions. The recruitment and evaluation of individuals in the comparison group took place at a later time than that of children with CZS. All parents or legal guardians voluntarily signed a term of informed consent. This study was approved by the Institutional Review Board of the Gonçalo Muniz Institute, Oswaldo Cruz Foundation (IGM-FIOCRUZ) (CEP protocol no. 1.935.854/2016).

### Procedures and assessments

Swallowing function was investigated through clinical and instrumental evaluations. Initially, we adopted part of a protocol elaborated to evaluate pediatric dysphagia (*Protocolo de Avaliação Clínica da Disfagia Pediátrica, PAD-PED)*^(16)^ and elaborated a checklist of clinical signs indicative of laryngotracheal penetration/aspiration in pediatric populations^(17)^. Second-stage evaluation consisted of the assessment of acoustic parameters using a Sonar Doppler device.

The PAD-PED protocol^(16)^ was used to assess aspects related to oral structures and food swallowing. With regard to oral structures, aspects (tonus, mobility and posture) related to the lips, tongue and cheeks were considered, as well as the condition of teeth (absence/presence), hard palate (normal/high-arched) and soft palate (normal/altered).

In functional examinations of swallowing food, events in the oral and pharyngeal phases were evaluated during the supply of non-viscous liquid (water) via baby bottle or plastic cup and homogeneous spoonable food (e.g. yogurt). In evaluations involving the ingestion of liquid from a bottle, the following aspects were evaluated: lip sealing, pausing, and sucking- swallowing-breathing coordination. With regard to the ingestion of liquid from a cup, aspects of adequate lip sealing, sipping movement, the pouring of liquid into the oral cavity, and escape through the lip commissures were observed. The evaluation of spoonable foods evaluated the upper lip contact with the spoon, tongue movement, escape through the lip commissures, and residual food in the oral cavity after swallowing^(16)^.

Oropharyngeal dysphagia (OPD) was classified based on the PAD-PED protocol^(16)^. Normal swallowing was considered in the absence of clinical signs of dysphagia; Mild dysphagia was defined as the presence of clinical signs resulting from inadequacies during feeding, which could be mitigated through postural adjustments, utensils, and food consistency. Moderate/severe dysphagia reflected changes in the pharyngeal phase of swallowing or changes in the oral phase that greatly impact the ability to maintain adequate nutrition and hydration. Children with this classification require food consistency restrictions and/or alternative complementary feeding. In severe dysphagia, oral feeding becomes impossible due to a high risk of presumed aspiration, thus requiring the exclusive use of alternative forms of feeding.

The following clinical indications of laryngotracheal penetration/aspiration were investigated: coughing during and/or after feeding, gagging, panting, labored breathing, and nasal regurgitation. These signs were selected from studies in the literature investigating children with cerebral palsy^(17)^. The presence or absence of each specific sign was recorded in accordance with food consistency (water or spoonable).

The instrumental evaluation of swallowing using a Sonar Doppler device was performed by ultrasound (model DF 7001 B, Medpej), via a single crystal disk transducer coupled to continuous-wave Doppler equipment connected to a Dell notebook (Intel Celeron M360 processor, Windows XP Professional).

In clinical evaluations the sequence of food presented to children was identical, i.e. liquid was initially offered by a parent/guardian in a bottle or cup (depending on each child’s typical preference at home), or using a spoon. All changes in food consistencies were preceded by three-minute intervals.

Clinical evaluations were carried out by two speech therapists (F1 and F2) who received identical instructions on how to judge each task and fill out forms, as well as training to ensure reliability. Overall agreement was measured by Cohen's Kappa Coefficient (κ). High agreement was observed after training in regards to most of the items evaluated: Structural and functional examinations (κ ꞊0.60, p<0.001); Evaluation of swallowing liquids (κ = 1, p<0.001); Clinical signs suggestive of laryngotracheal penetration/aspiration impairment during liquid ingestion (κ = 90, p <0.001); Evaluation of swallowing spoonable foods (κ = 1, p <0.001); Clinical signs suggestive of laryngotracheal penetration/aspiration impairment during the ingestion of spoonable foods (κ= 90, p <0.001).

To capture swallowing sounds, the children remained seated on their parents’/ guardians' lap in a comfortable position with no restraints around the neck. During the instrumental evaluation, liquid (water) and spoonable food (yogurt) were offered in a similar fashion as during clinical evaluations, with at least three swallows recorded for each type of consistency.

While attempting to capture swallowing sounds, reduction audio quality in the recordings occurred due to movement of the child's head, the child pulling or trying to grab the microphone, and crying and/or vocalizations heard pre-and/or post-swallowing. In these cases, examinations using Sonar Doppler were rescheduled.

Swallowing sounds were captured by a speech therapist trained in the use of a Sonar Doppler device (F3). The Sonar Doppler was placed on the right side of the neck, over the lateral region of the trachea just below the cricoid cartilage, the position described as the best site for cervical auscultation^(18)^. The beam of ultrasonic energy emitted by the transducer was positioned at an angle between 30º-60º. Contact® gel was used to decrease the dispersion of ultrasonic waves into the air and to increase body and echo transmission. Acoustic signals were recorded for later analysis. The Doppler equipment was adjusted to the lowest volume possible to minimize the interference of external noise.

Acoustic signals were recorded and voice analysis files were evaluated using VoxMetria software version 4.0^(19)^. Three variables were measured, as previously described by Abdulmassih et al.^(14)^: Initial Frequency (IF) of the sound wave - the frequency at the beginning of the acoustic process signal, measured in Hz; Peak frequency (PF) of the sound wave - the frequency of the highest point of displacement of the acoustic signal, measured in Hz; Initial intensity (II) - the initial intensity of the acoustic signal, varying from 10dB to 140dB; Peak intensity (PI) - amplitude of the sound signal, ranging from 10dB to 140dB; Swallowing time (T) - time elapsed, measured in seconds, from swallowing apnea to the descent of the larynx after post-swallowing expiration, completing the complete swallowing cycle from the beginning to the end of the acoustic signal.

The captured swallowing sounds were evaluated by a single speech therapist (F4), who remained blinded during the analysis of Sonar Doppler-generated swallowing sounds. Prior to beginning the study, the speech therapist received training emphasizing: (1) the definition of swallowing sounds, (2) examples of normal swallowing of liquid and spoonable food consistencies in five children and three adults; (3) filling in data on the collection form.

### Statistical analysis

All generated data were analyzed by a statistician who was blinded to data coding and not involved in any previous stages of the study. Statistical analyses were performed using SPSS 21.0 for Windows. Data were tested for normality using the Shapiro-Wilk test. Descriptive statistics (frequencies, means and dispersion measures) were calculated with respect to the acoustic parameters of peak frequency, duration and peak amplitude. The Student's t-test was used to assess differences between acoustic parameters related to swallowing liquid or spoonable food. Pearson's Chi-Squared and Fisher's Exact tests were applied to verify associations between categorical variables. Statistical significance was considered when p<0.05.

## RESULTS

A total of 90 children were evaluated, 45 with CZS and 45 age- and sex-matched controls exhibiting typical development. The mean ages of children with CZS and those in the comparison group were 26.69 ± 4.46 months and 26.62 ± 5.00 months, respectively. With regard to distribution by sex, females predominated 27(60%) in both groups. Children with CZS presented an average head circumference of 29.20 ± 1.98 cm. Most had calcifications 39(86.7%) and ventricular dilation 29(64.4%). Epilepsy was present in 28(62.2%) of the cases. Severe motor impairment was found in 40(88.9%) of the sample. The demographic and clinical characteristics of the children with CZS are shown in table 1.

**Table 1:** Clinical and demographic characteristics of 45 children with Congenital Zika Syndrome (CZS)

Characteristics	n (%)
Age in months (mean ± SD)	26.69 ± 4.46
Sex	
Male	18 (40)
Female	27 (60)
Gestational Age (weeks) (mean ± SD)	38.61 ± 1.74
Weight at birth (kg) (mean ± SD)	2.810 ± 517.64
Length at birth (cm) (mean ± SD)	38.61 ± 1.74
Head circumference at birth (cm) (mean ± SD)	29.20 ± 1.98
Neuroimaging abnormalities^*^	
Calcifications	39 (86.7)
Ventricular enlargement	29 (64.4)
Other findings (lissencephaly, volumetric reduction, agenesis of the corpus callosum, hydrocephalus)	25 (55.6)
Epilepsy	
Yes	28 (62.2)
No	16 (35.6)
GFMCS classification	
I-III	5 (11.1)
IV-V	40 (88.9)
Dietary consistency	
Liquid ingestion	45(100)
Spoonable food	45(100)
Solid food	13(28.9)

* Neuroimaging abnormalities evaluated in 39 children with CZS

Caption: SD = standard deviation; GMFCS = Gross Motor Function Classification System

Children with CZS demonstrated mild 13(28.9%) and moderate/severe 32(71.1%) OPD. All children in the comparison group presented normal swallowing ability. Clinical evaluations of oral structures revealed significant differences regarding most characteristics (p <0.05). Children with CZS frequently presented alterations related to resting lip posture 42(lips apart, 93.3%), changes in resting tongue posture 22(48.9%), increased tongue tonus 14(31.1%), decreased cheek tonus 34(75.6%) and high-arched hard palate 32(71.1%) (Table 2). Likewise, significant differences were found among the children with CZS and controls with respect to all items evaluated in clinical evaluations of swallowing liquid and spoonable food (Table 2). The majority 27(60%) of children with CZS ingested liquids from a baby bottle at home; clinical evaluations involving this form of swallowing liquid revealed poor lip seal in 36(66.7%), lack of coordination in sucking- swallowing-breathing in 36(66.7%), and the absence of pausing to breathe while sucking in 6(22.2%) of these children.

**Table 2:** Comparisons between children with congenital Zika syndrome and controls on clinical evaluations of oral structure and deglutition of liquids and spoonable food

Characteristics	Children with CZS (%)		Control group (%)		*p*
	n	%	n	*%*	
**Functional and Structural Examination**					
Lip closure	3	6.7	39	86.7	p<0.001
Normal lip tonus	11	24.4	40	88.9	p<0.001
Proper resting tongue posture	23	51.1	44	97.8	p<0.001
Normal lingual tonus	10	22.2	45	100	p<0.001
Cheek tonus	11	24.4	45	100	p<0.001
Teeth (presence)	43	95.6	45	100	0.153
Hard palate (normal morphology)	13	28.9	45	100	p<0.001
Soft palate (normal morphology)	43	95.6	45	100	0.153
**Liquid ingestion via baby bottle** ^*^					
Adequate lip sealing	9	33.3	-	-	-
Pausing to breathe while sucking	21	77.8	-	-	-
Proper sucking-swallowing-breathing coordination	9	33.3	-	-	-
**Liquid ingestion via cup**					
Adequate lip sealing	2	11.1	45	100	p<0.001
Sipping movement	4	22.2	45	100	p<0.001
Escape through the lip commissures	14	77.8	-	0	p<0.001
Pouring of liquid into the oral cavity	15	83.3	-	0	p<0.001
**Ingestion of spoonable food (yoghurt)**					
Lip/spoon contact	11	25.0	45	100	p<0.001
Lip sealing	8	18.2	45	100	p<0.001
Escape through the lip commissures	36	81.8	-	0	p<0.001
Tongue movement	22	51.1	45	100	p<0.001
Residual food in the oral cavity	38	86.4	45	0	p<0.001

χ^2^ test (p values ≤0.05)

* No members of the control group used baby bottles

Caption: CZS = Congenital Zika Syndrome

Appropriate lip closure was significantly associated with inefficient labial sealing (*x*^2^꞊0.004; p <0.05) and loss of liquid while swallowing due to insufficient lip closure (*x*^2^꞊0.021; p <0.05). With regard to swallowing spoonable food, insufficient lip closure was significantly associated with the following characteristics associated with spoon-feeding: lip/spoon contact (*x*^2^꞊0.002, p <0.05); incomplete upper lip contact (*x*^2^꞊0.000, p <0.005); leakage through the lip commissure (*x*^2^꞊0.024, p <0.05); presence of residuals following swallowing (*x*^2^꞊0.006, p <0.05). Resting tongue posture was also found to be significantly associated with the following aspects when swallowing spoonable foods: lip/spoon contact (*x*^2^꞊0.000; p <0.05); lip sealing (*x*^2^꞊0.000; p <0.05) and food leakage through the lip commissure (*x*^2^꞊0.000; p <0.05).

The evaluation of clinical signs indicative of laryngotracheal penetration/aspiration, revealed significant differences (p<0.05) in almost all characteristics assessed in children with CZS versus the comparison group. In children with CZS, higher frequencies of gagging 11(24.4%) and panting 11(24.4%) were found while swallowing liquid. With regards to spoonable food, the most prominent indicators were panting 11(26.7%) and labored breathing 9(22.2%) (Table 3).

**Table 3:** Comparisons between children with congenital Zika syndrome and controls on evaluation clinical signs indicative of pharyngeal phase impairment

Characteristics	Children with CZS (%)		Control group (%)		*p*
	n	%	n	*%*	
**Clinical signs indicative of pharyngeal phase impairment while drinking**					
Coughing during ingestion	8	17.8	0	0	0.003
Coughing after ingestion	10	22.2	0	0	0.001
Gagging	11	24.4	0	0	p<0.001
Panting	11	24.4	0	0	p<0.001
Labored breathing	5	11.1	0	0	0.021
Nasal regurgitation	1	2.2	0	0	0.315
**Clinical signs indicative of pharyngeal phase impairment (spoonable food)**					
Coughing during feeding	6	13.3	0	0	0.009
Coughing after feeding	5	11.6	0	0	0.019
Gagging	5	11.6	0	0	0.019
Panting	11	26.7	0	0	p<0.001
Labored breathing	9	22.2	0	0	0.001
Nasal regurgitation	1	2.3	0	0	0.304

χ^2^ test (p values ≤0.05)

Caption: CZS = Congenital Zika Syndrome

The instrumental assessment of swallowing using a Sonar Doppler device revealed non-significant shorter swallowing times regarding the ingestion of both liquid and spoonable food in children with CZS compared to the comparison group. No statistically significant differences were found in any of the acoustic parameters evaluated (Table 4).

**Table 4:** Analysis of swallowing sounds by sonar Doppler ultrasound in accordance with food/liquid consistency in children with CZS and controls

Liquid				Spoonable food				
	CZS	control		CZS	control			
	Mean (SD)	Mean (SD)	*p*	Mean (sD)		Mean (SD)		*P*
Age	26.69 ± 4.46	26.62 ± 5.00	.947	26.69 ± 4.46		26.62 ± 5.00		.947
IF	698.62 ± 59.24	699.24 ± 68.93	.964	690.13 ± 36.48		685.64 ± 31.15		.532
PF	1069.63 ± 22.20	1055.34 ± 61.36	.146	1057.76 ± 29.78		1056.88 ± 23.52		.876
II	54.87 ± 5.33	54.93 ± 6.20	.964	54.11 ± 3.2		53.70 ± 2.80		.532
PI	88.13 ± 2.15	86.98 ± 5.52	.197	87.19 ± 2.68		87.05 ± 2.08		.779
T	0.75 ± 0.28	0.79 ± 0.25	.441	0.81 ± 0.26		0.88 ± 0.17		.139

Caption: CZS = Congenital Zika Syndrome; SD = standard deviation; IF = initial frequency; PF = peak frequency; II = initial intensity; PI = peak intensity; T = time in seconds

## DISCUSSION

The present study investigated characteristics related to swallowing ability in children diagnosed with CZS and others with typical development. The swallowing ability of children with CZS was observed to be significantly impaired compared to the control group, as evidenced by clinical alterations both in the oral and pharyngeal phases of swallowing; however, no differences were seen in instrumentally-evaluated acoustic swallowing parameters.

All children with CZS presented OPD, and most had abnormalities in oral structures, which corroborates other findings in the literature using the same protocol in the clinical evaluation of similarly aged children with CZS^(20)^. The abnormalities observed herein in lip sealing and tongue and cheek tonus have been previously documented in children with CZS^(7,8,20)^, as well as in children with cerebral palsy (CP)^(21,22)^. These alterations have been linked to an impaired swallowing ability with regard to liquids and pasty foods, leading to difficulties in oral control, oral residues and loss of food/liquid through lip commissures^(23)^.

The lack of lip sealing combined with weakness and incoordination of tongue movements, as described in children with CP^(24)^, also interferes with the maintenance of intraoral pressure, possibly facilitating the premature escape of the bolus into the pharynx^(25)^. In addition, decreased cheek tonus in association with an impaired mobility and tonus of the tongue may promote the retention of residues in the oral cavity, as verified by a previous study involving babies diagnosed with CZS^(7)^. A high frequency of alteration in the palate was observed, indicating that this may be a common characteristic in children with CZS^(8,9)^, in contrast to fissures in the soft palate, which was not very prevalent^(20)^.

Changes in oral structures seen in children with CZS contributed to the impaired use of utensils during feeding, leading to the use of a baby bottle in most of the affected children studied herein. In children with CP, a higher prevalence of the use of bottles and training cups^(26)^ is probably associated with decreased tonus of the lips, which results in difficulties in grasping cups and sipping movements due to a lack of muscle strength; thus, liquids may be poured into the oral cavity when using a cup.

We found that children with CZS presented significant changes in most clinical signs related to laryngotracheal penetration/aspiration. A high percentage of signs described as initial symptoms of dysphagia have been reported in babies with CZS, such as coughing, gagging, breathlessness and labored breathing with effort^(7,20)^. Herein, the frequency of clinical signs indicative laryngotracheal penetration/aspiration varied in accordance with different types of food. Studies involving children with neurological impairments have described coughing during the ingestion of liquids^(27)^, which was also frequently found among the children with CZS studied herein. In addition, the presence of these signs represents a risk factor for aspiration and lung disease^(23,28)^.

Therefore, alterations found in the oral and pharyngeal phases of swallowing in children with CZS, which may have occurred due to the spectrum of this syndrome^(4-6)^, can impact both intervention strategies for speech therapy in CZS children, as well as the planning of public policies aimed at monitoring affected children.

No differences were seen between children with CZS and controls with regard to the acoustic parameters evaluated by the Doppler method in both liquid and homogeneous spoonable food consistencies. However, differences, such as those observed in studies involving adults with neurological disorders^(29)^ were expected between these groups considering the presence of clinical signs suggestive of laryngotracheal penetration/aspiration in children with CZS. It is important to note that our findings cannot be compared to other studies employing a similar Sonar Doppler technique, as the literature is lacking in this type of data in children with neurological disorders.

Considering the novelty of CZS syndrome and that associated clinical manifestations continue to be determined (4,5,8,15), the results of the present study are important to direct intervention strategies in Speech-Language Pathology, as well as for the planning of public policies aimed at minimizing deficits related to dysphagia and improving the quality of life of children with CZS.

### Limitations

The present study suffers from some limitations. First, although the sample size was identical in both groups, with larger numbers than other studies with similar objectives^(7)^, it may have been insufficient to reveal differences in acoustic parameters evaluated by the Doppler method used herein. Considering that the sample consisted of children with severe brain impairment and dysphagia, despite our expectation of significant differences between the groups, it is possible that acoustic parameters evaluated herein are not capable of revealing the expected distinctions; i.e. perhaps this technique is more useful in comparisons of the evolution of swallowing in children undergoing therapy, rather than providing a point of comparison between those with and without oral structural changes.

It is also important to consider the homogeneous nature of the present sample: children with microcephaly who present significant brain impairment. As such, any attempts to generalize the present findings should be made with caution, primarily because prenatal infection with the Zika virus can be considerably variable phenotypically. Lastly, as each child studied had a particular dietary pattern, resulting in non-uniform volumes of food or liquid offered, it must be acknowledged that differences in acoustic parameters during swallowing may result from both the volume of the bolus^(13)^ as well as viscosity^(30)^.

## CONCLUSION

The present study found that children with CZS present oropharyngeal dysphagia and alterations in the oral and pharyngeal phases of swallowing as compared to healthy subjects. These observed impairments in the mobility and tonus of oral structures contributed to difficulty in the oral phase of swallowing. In addition, the clinical signs indicative of laryngotracheal penetration/aspiration noted herein may lead to increased risk of respiratory infection. While no differences were seen in the acoustic parameters between the studied subjects and typical children, additional study will be necessary to determine the suitability of using a Sonar Doppler device in the assessment of swallowing in children with neurological disorders.
